# Potential Role of the Retrotrapezoid Nucleus in Mediating Cardio-Respiratory Dysfunction in Heart Failure With Preserved Ejection Fraction

**DOI:** 10.3389/fphys.2022.863963

**Published:** 2022-04-12

**Authors:** Camilo Toledo, Domiziana Ortolani, Fernando C. Ortiz, Noah J. Marcus, Rodrigo Del Rio

**Affiliations:** ^1^ Laboratory Cardiorespiratory Control, Department of Physiology, Pontificia Universidad Católica de Chile, Santiago, Chile; ^2^ Centro de Excelencia en Biomedicina de Magallanes (CEBIMA), Universidad de Magallanes, Punta Arenas, Chile; ^3^ Mechanisms of Myelin Formation and Repair Laboratory, Facultad de Ciencias de Salud, Instituto de Ciencias Biomédicas, Universidad Autónoma de Chile, Santiago, Chile; ^4^ Department of Physiology and Pharmacology, Des Moines University, Des Moines, IA, United States; ^5^ Centro de Envejecimiento y Regeneración (CARE), Pontificia Universidad Católica de Chile, Santiago, Chile

**Keywords:** heart failure, chemoreflex control, autonomic control, breathing disorders, chemoreceptors

## Abstract

A strong association between chemoreflex hypersensitivity, disordered breathing, and elevated sympathetic activity has been shown in experimental and human heart failure (HF). The contribution of chemoreflex hypersensitivity in HF pathophysiology is incompletely understood. There is ample evidence that increased peripheral chemoreflex drive in HF with reduced ejection fraction (HFrEF; EF<40%) leads to pathophysiological changes in autonomic and cardio-respiratory control, but less is known about the neural mechanisms mediating cardio-respiratory disturbances in HF with preserved EF (HFpEF; EF>50%). Importantly, it has been shown that activation of the central chemoreflex worsens autonomic dysfunction in experimental HFpEF, an effect mediated in part by the activation of C1 catecholaminergic neurons neighboring the retrotrapezoid nucleus (RTN), an important region for central chemoreflex control of respiratory and autonomic function. Accordingly, the main purpose of this brief review is to discuss the possible role played by activation of central chemoreflex pathways on autonomic function and its potential role in precipitating disordered breathing in HFpEF. Improving understanding of the contribution of the central chemoreflex to the pathophysiology of HFpEF may help in development of novel interventions intended to improve cardio-respiratory outcomes in HFpEF.

## Introduction

Heart failure (HF) is a complex and heterogeneous clinical condition that affects an estimated 64 million people worldwide (Groenewegen et al., 2020). HF in its various forms is generally characterized by a mismatch between tissue oxygen demand and supply due to impaired cardiac function. HF is classified according to the left ventricular ejection fraction (EF) as either HF with reduced EF (HFrEF; EF<40%) or HF with preserved EF (HFpEF; EF>50%) (Ponikowski et al., 2016). HFrEF is often observed following ischemic events, myocardial injury, and with coronary artery disease, and/or dilated cardiomyopathy or other events that lead to reduced cardiac contractility (Murphy et al., 2020). In contrast, HFpEF is generally characterized by elevated left ventricular stiffness during diastole which impairs ventricular relaxation (Pfeffer et al., 2019). Importantly, HFpEF incidence (≥ 50% in patients with HF) is increasing and is associated with substantial morbidity and mortality (Groenewegen et al., 2020).

Although, while HFrEF patients had benefit from complex pharmacological schemes and electronic-assisted devices for outcome improvements, these approaches showed low salutary potential when used in patients with HFpEF. Indeed, the complexity and heterogeneity of HFpEF may be one key reason for the poor track record of HFpEF clinical trials aiming to improve breathing disturbances in these patients. Thus, a more robust classification of the wide HFpEF phenotypic spectrum, particularly with a thoroughly characterization of chemoreflex drive, may help not only to improve HFpEF clinical management but also may shed lights into future therapeutic developments. In addition, there is an urgent need for new experimental HFpEF models that better reflects some of the specific pathological hallmarks of each (sub)type of human HFpEF for deeper understanding of the pathophysiological mechanisms involved in disease progression/maintenance (Conceição et al., 2016).

While there is conclusive evidence that demonstrates activation of the sympathetic nervous system (SNS) in HFrEF related with a chronically potentiated chemoreflex activity, there is limited information regarding chronic SNS activation in HFpEF (Verloop et al., 2015). However, both preclinical and clinical studies have shown a positive relationship between elevated SNS activity and the development of cardiac dysfunction (Verloop et al., 2015), and several studies show that experimental rat model that recapitulates the pathophysiology of HFpEF, display cardiac autonomic imbalance characterized by an increased sympathetic component (Toledo et al., 2017a; Andrade et al., 2019). Importantly, enhanced cardiac sympathetic tone is linked to arrhythmia incidence which may lead to increased risk of decompensation and mortality in HFpEF patients (Reddy et al., 2020).

In addition to autonomic dysfunction, sleep disordered breathing (SDB) (both central and obstructive) is highly prevalent in HFpEF patients and is associated with poor prognosis and increased mortality (Parati et al., 2016). SDB refers to a number of breathing disruptions that occur during sleep which elicit several physiological insults that worsen HF progression like arterial blood gas fluctuation, low tissue oxygen delivery, tonic/episodic increases in sympathetic discharges, neurohormonal activation, inflammation, and oxidative stress (Borrelli et al., 2019; Reddy et al., 2020). Importantly, autonomic nervous system (ANS) dysregulation has been extensively explored in SDB and has been recognized as one of the most important pathogenetic mechanisms for cardiovascular consequences (Lombardi et al., 2019). Thus, treatments targeting SDB in HFpEF may help pave the avenue to improve both, ANS and cardiovascular function being the outcome a reduction in disease progression.

Importantly, both cardiac autonomic imbalance and disordered breathing patterns in HF have been linked to altered chemoreflex function (Toledo et al., 2017b). Indeed, increased chemosensitivity to hypoxia and/or hypercapnia is an adverse prognostic factor in HF (Giannoni et al., 2009). In HFrEF, enhanced sympathetic activity and disordered breathing are closely related to increases in carotid body (CB) chemoreflex responses to hypoxia (Ponikowski et al., 1999; Del Rio et al., 2013). Conversely, central chemoreflex sensitivity is augmented in animal models of HFpEF where it is associated with enhanced cardiac sympathetic tone and irregular breathing patterns (Toledo et al., 2017a). Importantly, Kristen et al. showed that activation of central chemoreceptors elicits a ∼30% increase in renal sympathetic nerve activity in HF rats compared to healthy animals (Kristen et al., 2002). It is worth mentioning that this study was performed in baroreceptors and peripheral chemoreceptors denervated anesthetized rats (Kristen et al., 2002). Recently, we found that partial ablation of rostral ventrolateral medulla (RVLM) pre-sympathetic neurons abolishes the deleterious effects of central chemoreflex activation in HFpEF rats (Andrade et al., 2019; Toledo et al., 2019). Notably, it has been shown that activation of chemoreflex pathways plays a fundamental role in regulating tonic activity of RVLM pre-sympathetic neurons (Guyenet, 2014). Taken together, the current body of evidence strongly supports a role for central chemoreceptors in the development/maintenance of higher chemoreflex sensitivity, disordered breathing, and cardiac autonomic imbalance in the setting of HFpEF. The focus of this brief review is to discuss the potential mechanisms involved in the activation of central chemoreflex pathways in HFpEF and discuss how they may be linked to higher sympathetic outflow and disordered breathing patterns.

### Pathophysiology of HFpEF

HFpEF is characterized in part by left ventricular diastolic dysfunction. Ventricular filling is progressively impaired as a result of slow left ventricular relaxation and increased left ventricular stiffness (Pfeffer et al., 2019). This results in increased cardiac filling pressures at rest associated with a progressive decline in the passive properties of the left ventricle (Pieske et al., 2019). Other factors also contribute to the overall pathophysiology of the disease including ventricular desynchrony, increased left atrial pressure, pulmonary congestion, and atrial fibrillation (Qin and Heist, 2020). Many of these abnormalities are not present at rest and manifest only during cardiac stress (i.e., exercise) or during decompensation (Houstis et al., 2018; Lam et al., 2018). The pathophysiological heterogeneity that exists between patients with HFpEF is an important reason that effective therapies are yet to be developed (Lam et al., 2018). It has been proposed that interactions between autonomic function and control of breathing in the setting of HF could mediate, at least in part, disease progression/maintenance (Toledo et al., 2017b). Therefore, increasing collective knowledge of the mechanisms associated with autonomic imbalance and disordered breathing patterns in HFpEF may inform treatment strategies and ultimately improve disease outcomes.

### Disordered Breathing

The presence of disordered breathing (e.g., Cheyne-Stokes respiration, periodic breathing, apneas, and hypopneas) is associated with increased mortality in HF patients (Reddy et al., 2020). Most studies addressing the pathophysiological mechanisms and clinical implications of disordered breathing in HF have been done in HFrEF patients (or animal models of HFrEF) with fewer studies addressing HFpEF patients. Yet, sleep-related breathing disorders are highly prevalent in HFpEF and are thought to negatively impact autonomic and metabolic homeostasis (Baniak and Chasens, 2018). Indeed, it has been shown that disordered breathing patterns were present in ∼75% of HFpEF patients (Bitter et al., 2009). Furthermore, it has been suggested that patients with HFpEF and concomitant sleep-disordered breathing may be at higher risk for poor outcomes compared to those without breathing disorders (Baniak and Chasens, 2018). With respect to the potential relationship between disordered breathing and prognosis, disordered breathing may exacerbate tonic increases in sympathetic outflow in HFpEF patients due to intermittent stimulation of chemoreflex pathways and further increases in sympathetic activity. Further studies are needed to determine the role breathing disorders play in the progression of HFpEF and to identify the underlying mechanisms.

### Autonomic Imbalance

Chronic activation of the SNS and inhibition of the parasympathetic nervous system (PNS) are associated with poor outcomes in HFpEF (Verloop et al., 2015). While acute SNS activation provides inotropic support to the failing heart by increasing stroke volume and inducing peripheral vasoconstriction to maintain perfusion pressure, chronic SNS activation becomes maladaptive and accelerates disease progression, and increases mortality in HFpEF patients (Verloop et al., 2015). Several mechanisms could account for autonomic imbalance in HFpEF including, but not limited to, pathological activation of the renin–angiotensin system (Negi et al., 2015), chronic inflammation (Díaz et al., 2020a), elevated systemic oxidative stress (Negi et al., 2015), and cardiometabolic dysfunction (Savji et al., 2018). Importantly, all of these putative mechanisms also induce cardiac structural abnormalities which negatively impact cardiac function, creating a positive feedback loop that promotes SNS hyperactivation (Lam et al., 2018). In contrast to what is known about the SNS in the pathophysiology of cardiac failure, less is known about the parasympathetic nervous system in HFpEF. Parasympathetic withdrawal has been reported in both experimental and human HFpEF resulting in decreased heart rate variability and diminished sensitivity of the baroreflex (Phan et al., 2009). Unfortunately, no studies have comprehensively addressed the mechanisms associated with parasympathetic inhibition in HFpEF nor the potential therapeutic effect of parasympathetic modulation.

### Potential Role of Chemoreflexes in Driving Cardiorespiratory Dysfunction in HF

The carotid body (CB) chemoreceptors are the main peripheral arterial chemoreceptor in humans, sensing a wide variety of blood borne stimuli (i.e., PaO2, PaCO2, pH, blood flow, and temperature). Upon activation, the CB chemoreflex drives a highly coordinated cardiorespiratory response characterized by increased ventilation and sympathetic nerve activity (Schultz et al., 2007). In experimental HFrEF, increased tonic CB activity contributes to tonic increases in sympathetic outflow, higher arrhythmia incidence, and disordered breathing patterns (Del Rio et al., 2013). These results strongly support the notion that peripheral chemoreceptors play a pivotal role in the development and maintenance of cardio-respiratory disorders in ischemic HF. In contrast to HFrEF, little is known about the contribution of chemoreceptors in the pathophysiology of HFpEF, however there is some evidence that important physiological changes in central chemoreceptor activity play a role in the sympatho‐vagal imbalance and cardiorespiratory dysfunction observed in HFpEF (Toledo et al., 2017a; Díaz et al., 2020b).

Kristen et al. showed exaggerated increases in renal sympathetic nerve activity in response to hypercapnic stimulation in non-ischemic HF rats Kristen et al. (2002), and recent studies from our lab show that HFpEF rats have enhanced ventilatory chemoreflex response to hypercapnia (without changes in the hypoxic ventilatory response) combined with hyper-activation of brainstem pre-sympathetic neurons (Toledo et al., 2017a). Furthermore, episodic stimulation of retrotrapezoid nucleus (RTN) central chemoreceptor neurons exacerbates cardiac autonomic imbalance and disordered breathing in HFpEF rats (Díaz et al., 2020b). These observations have important implications for cardiac dysfunction during sleep-disordered breathing as an exaggerated chemoreflex response would be expected to add stress to the failing heart.

### Potential Integrative Role of the Retrotrapezoid Nucleus in Cardiorespiratory Regulation in HFpEF

Central chemoreception is characterized by changes in neuronal excitability of discrete populations of neurons in the brain in response to changes in the pH of cerebral spinal fluid. These neurons regulate activity of neurons in respiratory centers of the brainstem to adjust pulmonary ventilation in a manner necessary to maintain PaCO_2_/pH homeostasis (Guyenet et al., 2019). There are numerous regions in the brain that have been shown to be CO_2_/H^+^ sensitive; however, one of the main central chemoreceptor areas are found within the RTN, on the ventral surface of the medulla oblongata (Guyenet et al., 2019). Some studies have shown that this area accounts for ∼90% of the total central chemoreflex drive during exposure to hypercapnia (Kumar et al., 2015), and others suggest that the RTN may serve as an activity integration site due to the convergence of chemosensory information from other central and peripheral chemosensitive areas.

The RTN is defined as a set of neurons that: express the isotype 2 of vesicular glutamate transporter (VGLUT2), express the transcription factor Phox2b, lack catecholaminergic biosynthetic enzymes, and express the Neurokinin 1 receptor (NK1), and Neuromedin B (Shi et al., 2017). RTN chemoreceptor neurons are stimulated by [H^+^] through the activation of both a proton-activated G protein-coupled receptor (GPR4) and a proton-modulated potassium channel (TASK-2) (Kumar et al., 2015). During activation, RTN neurons stimulate reflex increases in pulmonary ventilation to mitigate hypercapnia, and also trigger cardiovascular changes (Kanbar et al., 2010).

**FIGURE 1:**
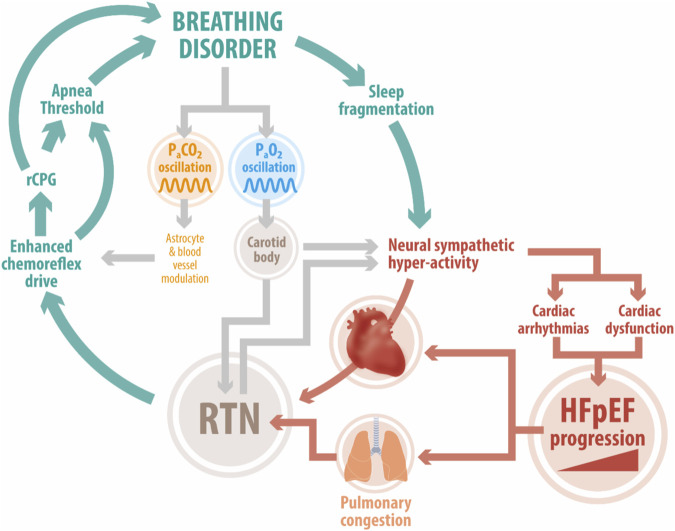
Deleterious positive feedback loop in HFpEF. Enhanced chemoreflex drive is a pathophysiological hallmark in HFpEF patients. Studies in experimental HFpEF show that acute activation of the central chemoreflex exacerbates elevated cardiac sympathetic tone, disordered breathing, and cardiac dysfunction. The RTN, an important locus of central chemoreception, may play a role in development/maintenance of disordered breathing patterns (i.e., periodic breathing, sleep apnea). Acute fluctuations in P_a_CO_2_ affect RTN and carotid body chemoreceptor activity which together stimulate increases in sympathetic outflow and pulmonary ventilation. This in turn hastens the development of cardiac arrhythmias and cardiac dysfunction. In addition, breathing disorders can trigger sleep fragmentation which further exacerbates sympathoexcitation and stresses the failing heart. Abbreviation: HFpEF, heart failure with preserved ejection fraction; RTN, Retrotrapezoid nucleus; RPG, respiratory pattern generator; P_a_CO_2_, arterial pressure of CO_2_; P_a_O_2_, arterial pressure of O_2_; CBs, carotid bodies.

Importantly, the RTN is point of convergence of polysynaptic excitatory inputs from other central and peripheral sites, including the CBs and acts as an integration center (Figure 1) (Guyenet et al., 2018). The activity of peripheral and central chemoreceptors has interdependent feedback regulation on breathing in order to minimize arterial PCO_2_ fluctuations. Indeed, it has been shown that CB stimulation/inhibition modulates central chemoreceptors sensitivity to CO_2_/H^+^ (Smith et al., 2015). Whether tonic CBs chemosensory activity may contribute to heightened RTN-mediated chemoreflex drive in HFpEF remains to be determined.

In non-ischemic HF patients and animal models, impaired diastolic function and stiffened myocardium increases the loading of the right ventricle leading to pulmonary vascular pressure increases and congestion (Angeja and Grossman, 2003). Congestion in turn has the potential to stimulate juxta-capillary receptors (type J receptor) located within the lung tissue (Paintal et al., 1973). Neural impulses from pulmonary J receptors are transmitted via afferent pulmonary vagal C-fibers that project to respiratory centers eliciting brief central apneas followed by a rapid shallow breathing (Kubin et al., 2006). Furthermore, pulmonary congestion could also trigger maladaptive respiratory responses to harmful distention of the lungs due to alterations in the function of both slow adapting and rapid adapting stretch receptors (SAR and RAR, respectively) situated in the lung parenchyma (Kubin et al., 2006). Importantly, RTN neurons receive inhibitory inputs from lung stretch afferents via direct projections from the nucleus tractus solitarius and the CPG, as a mechanism of feedback to reduce the influence of central chemoreceptors on ventilation under specific circumstances (Moreira et al., 2007). Whether maintained/chronic high activity levels rising from pulmonary sensory afferents during HFpEF may contribute to central chemoreceptor neuroplasticity and breathing dysregulation remains to be determined.

### RTN and Control of Breathing

**FIGURE 2:**
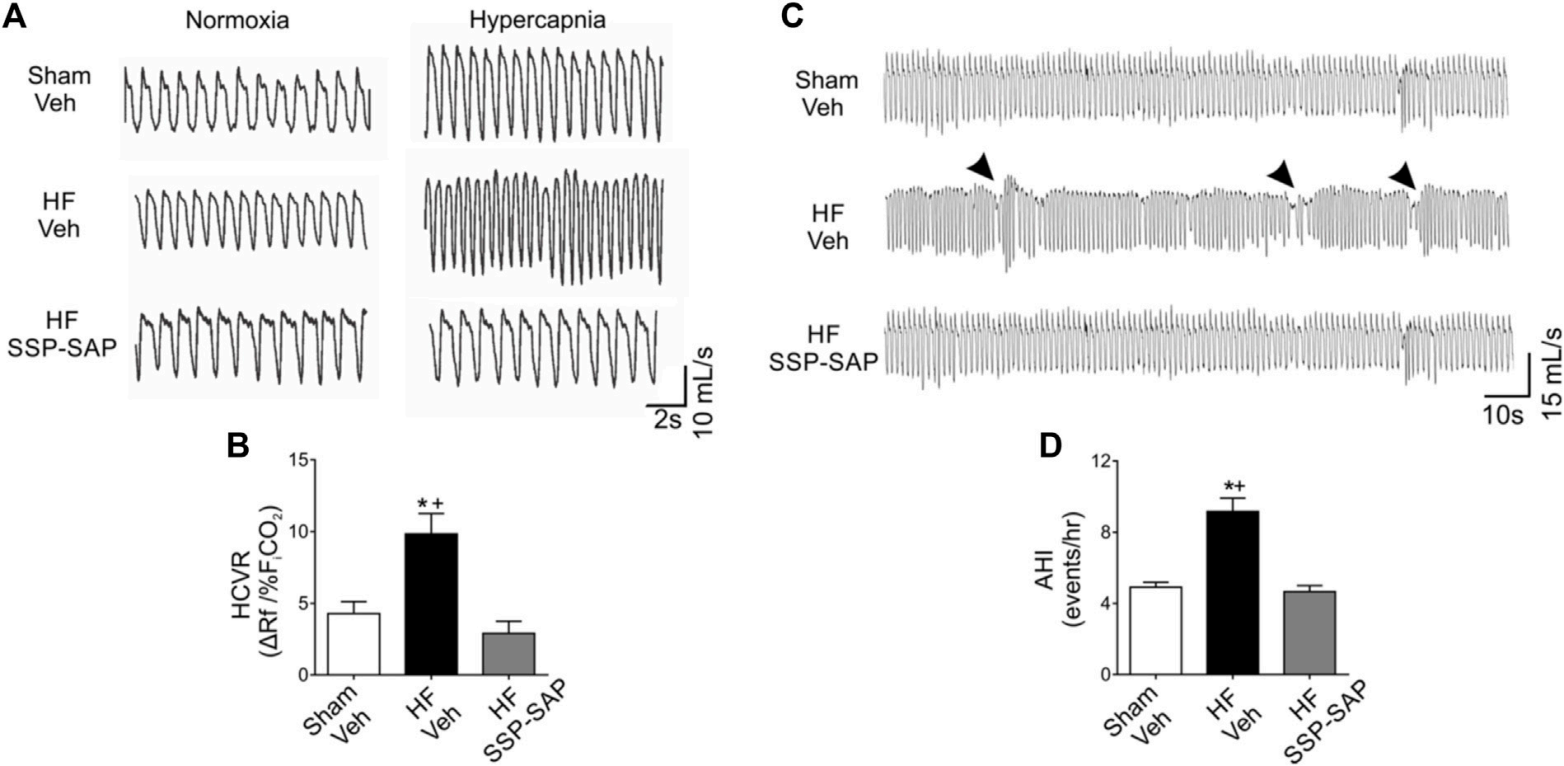
Partial ablation of RTN neurons blunts the hypercapnic ventilatory response and decreases incidence of breathing disorders in HFpEF rats. **(A)** Representative traces of ventilation during normoxia (F_i_CO_2_ 0.03%), and hypercapnia (F_i_CO_2_ 7%) in one rat per group. **(B)** Summary data of hypercapnic ventilatory response (HCVR). Note that the increased HCVR in HF rats is abrogated by partial ablation of RTN neurons using substance P-conjugated saporin (SSP-SAP) toxin. **(C)** Representative traces of ventilation in normoxia at rest. Arrows depict breathing disturbances (i.e., apneas/hypopneas). **(D)** Partial ablation of RTN neurons decreases the apnea/hypopnea index (AHI) in HF rats. Modified from Am J Physiol Lung Cell Mol Physiol. 318: L27–L40, 2020.

During subtle changes in cerebrospinal fluid CO_2_/H^+^ content, RTN chemoreceptor neurons send direct excitatory inputs to respiratory control centers to initiate breathing (Guyenet et al., 2019). Less is known about how RTN chemoreceptor neurons regulate the respiratory pattern under eupneic conditions. Interestingly, RTN neurons are able to respond to changes in pH independently of the degree of activation of rhythmic neurons located in the central pattern generator suggesting that RTN neurons could contribute to the development of altered breathing patterns following oscillations in cerebrospinal fluid [H^+^] content (i.e., spontaneous apneas/hypopneas) (Feldman and Del Negro, 2006). In addition, partial ablation of RTN neurons in healthy rats substantially increases apneic threshold suggesting that tonic activity from RTN neurons contributes to respiratory regulation in the absence of hypercapnia (Takakura et al., 2008). Importantly, partial ablation of RTN neurons decreases apneas/hypopneas index and improves breathing pattern regularity at rest in HFpEF rats (Figure 2). Furthermore, RTN neurons regulate breathing via a sleep-state dependent mechanism (Burke et al., 2015a). Therefore, alterations in RTN neuron activity may also contribute to sleep disruption in pathological conditions such as HF.

Several cellular mechanisms in the RTN may be involved in the development of respiratory alterations which are not necessarily limited to RTN chemoreceptor neurons but also can include RTN astrocytes and modulation of cerebral vascular function. It has been shown that astrocytes residing within the RTN also serve as “chemoreceptors” since they can be stimulated by CO_2_/H^+^ via inhibition of a pH-sensitive inwardly rectifying K^+^ channel and release one or several putative molecules, which can regulate RTN neuron activity (Gourine et al., 2010). Indeed, RTN astrocytes release ATP in a Ca^2+-^dependent process through connexin-26 during hypercapnia (Huckstepp et al., 2010). Importantly, purinergic modulation of RTN neuronal excitability by P2 receptors has also been described (Mulkey et al., 2006). While it is well established that increases in CO_2_/H^+^ cause vasodilation in cerebral blood vessels, it has been shown that purinergic signaling via P2Y2/4 receptors contributes to arteriolar tone in the RTN preventing CO_2_/H^+^-induced dilation and allowing for hypercapnia to stimulate increases in ventilatory drive (Hawkins et al., 2017). Applications of selective purinergic P2X receptor blockers into the RTN modify the respiratory pattern under normocapnic conditions, suggesting that purinergic signaling within the RTN during eupnea may modulate respiratory control (Barna et al., 2016). Despite these findings, the contribution of RTN astrocyte/blood vessels to respiratory control during normocapnic conditions merits further study, as does the role of RTN astrocytes in chemoreflex and resting breathing alterations in HFpEF.

### RTN and Autonomic Regulation

It has been suggested that hypercapnic stimulation can increase sympathetic outflow by activation of RVLM pre-sympathetic neurons (Guyenet, 2014). Indeed, in anaesthetized sino-aortic denervated and vagotomized rats a brief hypercapnic challenge (10% end-expiratory CO_2_) elicited a two-fold increase in lumbar sympathetic nerve discharge and this was associated with a ∼70% increase in RVLM activity (i.e. sympatho-excitatory neurons) (Moreira et al., 2006). Yet the mechanism responsible for the activation of RVLM neurons by hypercapnia is not known. Optogenetic activation of RTN neurons causes arousal as well as increases in sympathetic nerve activity and blood pressure in conscious rats (Abbott et al., 2013a), and stimulation of RTN chemoreceptor neurons after the lesion of spinally projecting catecholaminergic neurons has minimal effects on blood pressure and arousal (Burke et al., 2015b). These observations support the notion that increases in sympathetic drive during hypercapnia require the activation of catecholaminergic neurons following RTN activation. In addition, Abbott and colleagues demonstrated that RVLM‐C1 neurons play important roles in control of autonomic function and breathing since selective activation of these neurons increases ventilatory rate in healthy mice (Abbott et al., 2013b). Interestingly, reciprocal anatomical projections between the RTN and RVLM have previously been described (Rosin et al., 2006), but the presence of a direct excitatory input from the RTN chemosensitive neurons to RVLM pre-sympathetic neurons still awaits confirmation.

In HFpEF rats, partial lesion of RVLM-C1 neurons reduces the deleterious effects of central chemoreflex activation on cardiac autonomic imbalance, breathing disorders and cardiac function (Andrade et al., 2019; Toledo et al., 2019). Also, RTN chemoreceptor ablation in experimental HFpEF improves cardiac autonomic control and reduces chemoreflex-induced cardiac arrhythmias (Díaz et al., 2020b). Taken together, these results support the notion of a functional RTN-RVLM network that contributes to cardiorespiratory regulation and to the emergence of cardiorespiratory disorders in HFpEF. This exciting idea warrants future study to identify functional excitatory projections and to explore how they may be altered in the setting of HFpEF.

### Mechanisms Underlying Cardiorespiratory Alterations in HFpEF

As mentioned previously, central chemoreflex sensitivity is enhanced in HFpEF and is associated with sympatho-excitation and cardiorespiratory dysfunction. While the precise mechanism(s) responsible for central chemoreflex sensitization, autonomic imbalance, and respiratory disorders in HFpEF are not yet clearly established, many studies suggest that neuroinflammation and oxidative stress observed in autonomic brain regions play a crucial role in cardiac pathophysiology in HF independent of its etiology (HFrEF vs. HFpEF). Persistent/chronic neuroinflammation and oxidative stress create a complex positive feedback loop that induces and maintains elevated sympathetic activity in HFpEF mediated in part by central chemoreflex hypersensitivity. Indeed, high levels of reactive oxygen species (ROS) have been linked to sympatho-excitation in HF (Díaz et al., 2020a) and our lab has previously shown high levels of brainstem ROS in rats with HFpEF (Díaz-Jara et al., 2021). In addition, recent studies from our lab in which we delivered brain-targeted pharmacological inhibition of ER stress restored normal cardiorespiratory function in HFpEF rats (Díaz et al., 2021), suggesting an important link between neuroinflammation, oxidative stress, ER stress, and neuronal excitability in the brainstem.

Finally, as mentioned earlier, astrocytes contribute to ventilatory drive under hypercapnia through purinergic signaling (Wenker et al., 2012). Importantly, beyond the release of ATP, astrocytes have the potential to release a large variety of pro-inflammatory molecules such as cytokines (i.e., IL-1β, TNF-α) and chemokines (Sofroniew, 2015). Therefore, it is possible that intermittent fluctuations in PaCO_2_/H^+^ induces astrocyte-mediated release of pro-inflammatory cytokines in brainstem areas related to respiratory/sympathetic regulation which may contribute to enhanced SNS outflow and de-stabilize respiratory control. Understanding the complex interplay of the mechanisms involved in central chemoreflex hypersensitivity, increased sympathetic activity, and disordered breathing is an important step toward the development of novel interventions and improved clinical outcomes in HFpEF.

## Summary

The incidence and prevalence of HFpEF relative to HFrEF is rising at a rate of 10% every 10 years and will become the most common form of HF within the next decade (Groenewegen et al., 2020). There are currently no effective therapies for HFpEF due in part to a lack of knowledge of the precise mechanisms underlying this complex disease state. Enhanced central chemoreflex sensitivity, cardiac autonomic imbalance, and breathing disorders are all observed in experimental animals with non-ischemic HF with preserved EF. Moreover, activation of the chemoreflex pathway in HFpEF rats further compromises cardiac function through the activation of the sympathetic nervous system. RTN chemoreceptor neurons may serve as a nodal point for controlling both pulmonary ventilation and autonomic function in HFpEF. Future studies should focus on identifying cellular and molecular mechanisms underlying central chemoreflex alterations observed in HFpEF and how these alterations contribute to disease progression.
